# Barriers to cervical cancer screening faced by immigrant Muslim women: a systematic scoping review

**DOI:** 10.1186/s12889-023-17309-9

**Published:** 2023-11-30

**Authors:** Yusi Riwayatul Afsah, Noriyo Kaneko

**Affiliations:** 1 School of Nursing, Universitas Muhammadiyah Yogyakarta, Bantul, Yogyakarta, 55183 Indonesia; 2 Graduate School of Nursing, Global and Community Health Nursing, Nagoya City University, Nagoya, 467-0001 Japan

**Keywords:** Muslim, Cervical cancer, Barriers, Screening

## Abstract

**Background:**

Uptake for cervical cancer screening (CCS) is extremely low among immigrant women, particularly Muslim women, because of barriers related to religious values, beliefs, and fatalism. This scoping review aimed to summarize and analyze the findings of previous studies regarding perceived barriers to CCS among Muslim immigrant women.

**Methods:**

A search of electronic databases of peer-reviewed articles, including MEDLINE, CINAHL, PubMed, and Scopus was conducted. The following criteria were used for the selection of the articles: (a) the study population consisted of immigrant Muslim women, (b) CCS barriers were the main focus of the study, (c) the articles were original research articles, (d) the research was conducted within the last 10 years, and (d) the study was reported in English language.

**Results:**

Barriers included sociodemographic factors, economic, language, cognitive, and emotional reactions. The healthcare system was classified as a community barrier, whereas culture and religion were categorized as social barriers. Beliefs that becoming ill and dying is the will of Allah and that health problems are a punishment from God were considered to be major barriers to CCS among immigrant Muslim women.

**Conclusion:**

Access to health service centers and CCS among Muslim immigrant women is challenging. Information dissemination by health care workers is needed to increase awareness of CCS and access to CCS service points among immigrant Muslim women. Physician recommendations to attend CCS also play an important role.

## Background

Globally, cervical cancer is the fourth most common cancer among women, with approximately 90% of new cases and deaths in 2020 mostly occurring in low- and middle-income countries [1]. Early detection of cancerous lesions via cervical cancer screening (CCS) leads to a positive diagnosis and a high chance of cure. According to the International Agency for Research on Cancer, the CCS program has succeeded in becoming an effective strategy to reduce the incidence of the disease [2]. In developed countries, CCS is widely available. However, uptake for the screening is extremely low among immigrant women, including Muslim women, in Canada, the US, and Australia [2–4]. Regardless of religion or ethnicity, immigrant women face barriers to screening related to socioeconomic status, health insurance status, inadequate language skills, lack of awareness of screening test, difficulties in accessing health care services, cultural beliefs, and anxiety regarding screening test procedures [5]. These obstacles are exacerbated among immigrant Muslim populations who face additional barriers related to religious values, beliefs, and fatalism [6–8].

Interestingly, only a few studies have investigated CCS barriers among immigrant Muslim women, with inconsistent results. Some studies found that health care systems, cultural barriers, and religion can be obstacles; however, other studies did not identify these factors as obstacles to screening tests [2, 7–9]. Muslims represent a large proportion of the population in many countries, including non-Muslim-majority countries. Muslims are the fastest-growing religious group in the world with a projected 35% increase in the Muslim population over the next 20 years; specifically, the number of Muslim immigrant women requiring regular CCS is predicted to increase in Europe, Canada, and Japan [10].

To develop a CCS program that engages with Muslim women, it is necessary to identify the perceived barriers to screening among Muslim immigrants. Previous research undertaken in different countries identified different obstacles to CCS. Most studies have focused on barriers and predictors of cervical cancer uptake among immigrants in one specific country. Furthermore, many studies were conducted in countries with high immigrant populations in which immigrant support services were available. There is a paucity of review articles conducted in the past 5 years focusing on barriers to CCS among Muslim immigrants. A scoping review approach was chosen for this study to examine the overall results and compare the findings of the studies among different countries. The aim was to identify common Muslim-specific barriers to CCS that are not country-specific. A review focused on CCS barriers among Muslim immigrant women can provide an understanding of the obstacles faced, a more comprehensive identification of the existing challenges, and the review can identify gaps in the current knowledge to be addressed in future research.

## Methods

**Fig. 1:**
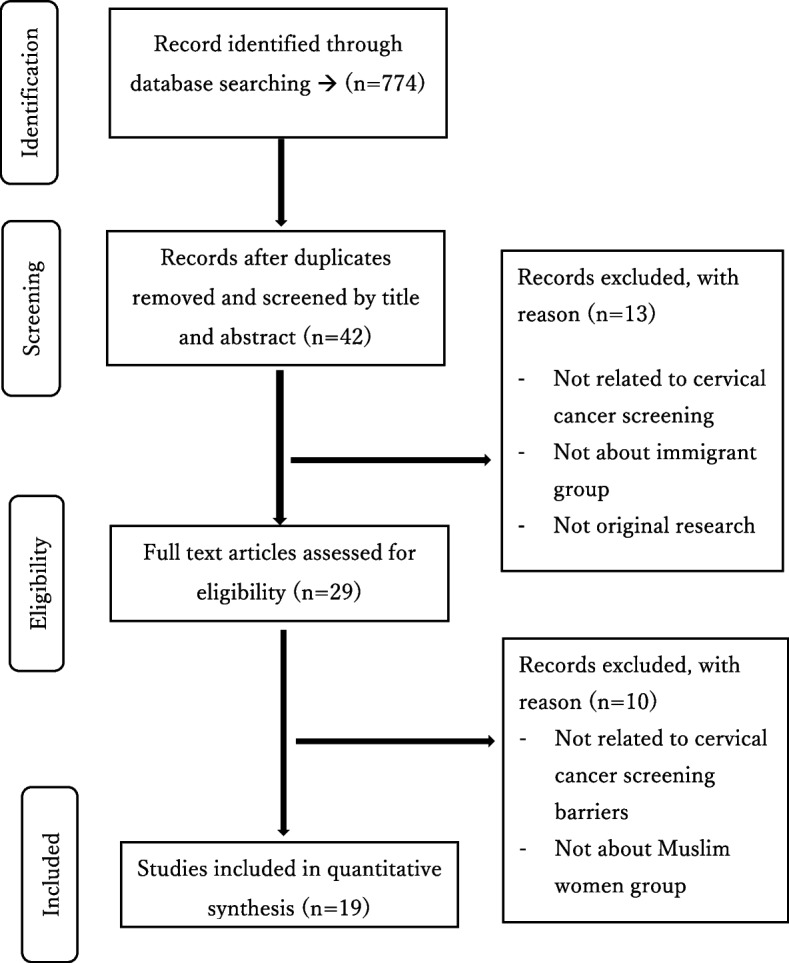
Flowchart for selection studies

This scoping review applied the methodological framework proposed by Arksey and O’Malley based on five implementation stages: 1) identifying the research questions; 2) identifying the relevant literature; 3) study selection; 4) mapping the data; and 5) summarizing, synthesizing and reporting the results [11]. Firstly, we identified our research question as follows: “What are the obstacles faced by immigrant Muslim women in CCS?”. Second, we searched the literature for evidence using various sources including electronic databases, main journal searches, and reference lists. Third, two reviewers applied the inclusion and exclusion criteria to all citations. Copies of the full articles were obtained studies that appeared to be representative and best suited to the research question. If the relevance of a study was not clear from the abstract, then the full article was obtained. A deadline was set, after which it was agreed that no further studies would be included in the analysis. The next stage involved making a final decision on the studies selected for review. Fourthly, a ‘data chart form’ using the Excel database program was created and information including author, publication year, study type, data source, location, and study population (size, age, sample) was recorded. Finally, we applied several analytic frameworks and thematic constructs to present a narrative of the existing literature. The article search and review process began on August 31, 2022. A flowchart of the selection process is shown in Fig. 1.

### Source of the data

We comprehensively and systematically searched the electronic databases of peer-reviewed articles for research published between 2012 and 2022. The review and analysis process were conducted between August 2022 and July 2023. The databases searched include MEDLINE, CINAHL, PubMed, and Scopus. A comprehensive Medical Subject Heading term and keywords in four major categories (Muslim, barriers, screening, cervical cancer) were used for the research (Table 1). Keywords were concatenated with the Boolean operator “OR” and all main components were concatenated with the Boolean operator “AND.”

**Table 1:** Search strategy. The article search and review process was conducted between 31 August 2022 and July 2023

Keyword for barriers: "barrier s"[All Fields] OR "barriers"[All Fields] OR ("factor"[All Fields] OR "factor s"[All Fields] OR "factors"[All Fields]) OR ("risk"[MeSH Terms] OR "risk"[All Fields]) OR "risk factor"[All Fields] OR ("prejudice"[MeSH Terms] OR "prejudice"[All Fields] OR "prejudices"[All Fields]) OR ("attitude"[MeSH Terms] OR "attitude"[All Fields] OR "attitudes"[All Fields] OR "attitude s"[All Fields]) OR ("issue"[All Fields] OR "issue s"[All Fields] OR "issues"[All Fields]) OR "self-conscience"[All Fields] OR "attitude of health personnel"[All Fields] OR ("attitude to health"[MeSH Terms] OR ("attitude"[All Fields] AND "health"[All Fields]) OR "attitude to health"[All Fields]) OR ("difficulties"[All Fields] OR "difficulty"[All Fields]) OR ("obstacle"[All Fields] OR "obstacles"[All Fields]) OR ("obstruct"[All Fields] OR "obstructed"[All Fields] OR "obstructing"[All Fields] OR "obstruction"[All Fields] OR "obstructions"[All Fields] OR "obstructive"[All Fields] OR "obstructs"[All Fields]) OR ("challenge"[All Fields] OR "challenged"[All Fields] OR "challenges"[All Fields] OR "challenging"[All Fields]) OR ("confront"[All Fields] OR "confrontation"[All Fields] OR "confrontational"[All Fields] OR "confrontations"[All Fields] OR "confrontative"[All Fields] OR "confronted"[All Fields] OR "confronting"[All Fields] OR "confrontive"[All Fields] OR "confronts"[All Fields]) OR "defy"[All Fields] OR ("mistrust"[All Fields] OR "mistrusted"[All Fields] OR "mistrustful"[All Fields] OR "mistrusting"[All Fields]) OR ("hinder"[All Fields] OR "hindered"[All Fields] OR "hindering"[All Fields] OR "hinders"[All Fields]) OR ("impediment"[All Fields] OR "impediments"[All Fields]) OR ("hurdle"[All Fields] OR "hurdles"[All Fields]) OR ("difficulties"[All Fields] OR "difficulty"[All Fields]) OR "defiance"[All Fields] OR "health knowledge attitudes practice"[All Fields] OR ("uncertainty"[MeSH Terms] OR "uncertainty"[All Fields] OR "uncertainties"[All Fields]) OR ("object"[All Fields] OR "object s"[All Fields] OR "objectness"[All Fields] OR "objects"[All Fields]) OR ("contest"[All Fields] OR "contestability"[All Fields] OR "contestable"[All Fields] OR "contestant"[All Fields] OR "contestants"[All Fields] OR "contestation"[All Fields] OR "contestations"[All Fields] OR "contested"[All Fields] OR "contesting"[All Fields] OR "contests"[All Fields]) OR ("question"[All Fields] OR "question s"[All Fields] OR "questionable"[All Fields] OR "questionables"[All Fields] OR "questionably"[All Fields] OR "questioned"[All Fields] OR "questioner"[All Fields] OR "questioners"[All Fields] OR "questioning"[All Fields] OR "questionings"[All Fields] OR "questions"[All Fields]) OR "health services accessibility"[All Fields] OR ("antagonists and inhibitors"[MeSH Subheading] OR ("antagonists"[All Fields] AND "inhibitors"[All Fields]) OR "antagonists and inhibitors"[All Fields] OR "inhibitors"[All Fields] OR "inhibitor"[All Fields] OR "inhibitor s"[All Fields]) OR ("roadblock"[All Fields] OR "roadblocks"[All Fields]) OR ("block"[All Fields] OR "blocked"[All Fields] OR "blocking"[All Fields] OR "blockings"[All Fields] OR "blocks"[All Fields]) OR ("pitfall"[All Fields] OR "pitfalls"[All Fields]) OR "communication barriers"[All Fields])) AND ((y_10[Filter]) AND (ffrft[Filter]) AND (english[Filter]))
Keyword for screening: "screening"[All Fields] OR "Mass Screening"[MeSH Terms] OR ("mass"[All Fields] AND "screening"[All Fields]) OR "Mass Screening"[All Fields] OR "early detection of cancer"[MeSH Terms] OR ("early"[All Fields] AND "detection"[All Fields] AND "cancer"[All Fields]) OR "early detection of cancer"[All Fields] OR "screen"[All Fields] OR "screenings"[All Fields] OR "screened"[All Fields] OR "screens"[All Fields] OR "Mass Screening"[All Fields] OR "Preventive test"[All Fields] OR "preventive investigation"[All Fields] OR "early diagnosis"[All Fields])
Keywords for cervical cancer: (("Cervical cancer"[All Fields] OR "Uterine cervical neoplasms"[All Fields] OR "cervical neoplasm"[All Fields] OR "Pap Smear"[All Fields] OR "Papanicolaou Test"[All Fields] OR "Pap test"[All Fields] OR "vaginal smears"[All Fields] OR "vaginal smear"[All Fields]) AND ("diagnosis"[MeSH Subheading] OR "diagnosis"[All Fields]
Keywords for Muslim "islam"[All Fields] OR "muslim"[All Fields] OR "muslims"[All Fields] OR ("islam"[MeSH Terms] OR "islam"[All Fields] OR "islamic"[All Fields] OR "islam s"[All Fields] OR "islamism"[All Fields]) OR ("islam"[MeSH Terms] OR "islam"[All Fields] OR "moslem"[All Fields] OR "moslems"[All Fields]) OR ("islam"[MeSH Terms] OR "islam"[All Fields] OR "islamic"[All Fields] OR "islam s"[All Fields] OR "islamism"[All Fields]) OR ("religion"[MeSH Terms] OR "religion"[All Fields] OR "religions"[All Fields] OR "religion s"[All Fields]))

### Study selection

Development-specific inclusion and exclusion criteria were used to screen out irrelevant articles. The following inclusion criteria were used:

- The research sample consisted of immigrant Muslim women
- CCS barriers were the research focus
- The study was an original research article
- The research was conducted within the last 10 years
- The study was reported in English

The following exclusion criteria applied:

- Study populations including temporary or refugee residents or illegal immigrants
- Studies that did not include Muslim women as participants
- Studies that were not related to CCS barriers
- Editorials, reviews, conference abstracts, guidelines, case reports, or papers only describing the study design.

Articles were selected in two steps. First, two independent reviewers (ARY, KN) completed the selection of titles and abstracts based on the inclusion and exclusion criteria. Secondly, the two independent reviewers completed full-text analyzes of the studies selected in phase one, based on the inclusion and exclusion criteria. Resolution of differences in ideas or views between the reviewers was achieved through discussion.

### Data extraction

The characteristics of each article were summarized and mapped in Microsoft Excel, including research title, name of author, year of publication, research objectives, research design, sources of data acquisition, research site, sample size, study population, participant characteristics (such as age, country of origin), and the obstacles identified. The articles were categorized based on the barriers identified in the research to develop research descriptions and perspectives, and create a thematic construction to display research findings that have been conducted in this area.

## Results

The electronic database search identified 774 peer-reviewed studies. Following the removal of duplicates (*n* = 732), title and abstract screening were performed for 42 articles, and 13 articles were excluded. A total of 29 articles were reviewed in full detail, resulting in the exclusion of 10 articles. Finally, 19 articles were selected for data extraction, analysis, and synthesis. A review of the reference list of the 19 selected articles was also conducted. In this study, gray literature was not included in the final review (Fig. 1).

### Selected studies characteristics

The characteristics of the selected studies are presented in Table 2. All 19 studies were conducted between 2012 and 2022. Most were conducted in the US (*n* = 7), followed by Canada (*n* = 3). The research methods used in the articles were qualitative (*n* = 9) and quantitative (*n* = 9). One study used a combination of qualitative and quantitative methods. Data collection methods and sources included focus groups, surveys (questionnaires, databases, internet-based), in-depth interviews, semi-structured focus group interviews, group interventions, and evaluations.

**Table 2:** Characteristics of selected studies

NO	Reference No	Author	Year	Study Type	Data Source	Location	Size	Age	Sample
1	[12]	Salman	2012	Quantitative	Survey questionnaire	US	50	18–50 +	Arab women
2	[8]	Wong et al	2013	Quantitative	Questionnaire	Selangor, Malaysia	231	25–53	Malay, Chinese, Indian
3	[13]	Padela et al	2014	Quantitative	Survey questionnaire	Chicago	254	21–65	Arab/Arab American, African American/Black, South Asian
4	[14]	Khan & Woolhead	2015	Qualitative	In-depth interviews	Dubai	13	≥ 18	Emirati women, South Asian
5	[15]	Marlow et al	2015	Qualitative	Interview	London	54	25–64	Indian, Pakistani, Bangladeshi, Caribbean, African, White British
6	[3]	Vahabi & Lofters	2016	Qualitative	Focus group interview	Canada	30	21–69	West Asian, South Asian
7	[16]	Zorogastua et al	2017	Qualitative and quantitative	Focus groups and individual questionnaires	New York City	140	18–70	Gambia, Guinea, Ivory, Mali, Morocco, Senegal, United States, other African countries
8	[7]	Lofters et al	2017	Quantitative	Database survey	Ontario, Canada	761,019	21–69	East Asia, Eastern Europe & Central Asia, Middle East & North Africa, South Asia, Sub-Saharan Africa
9	[6]	Gele et al	2017	Qualitative	Focus group discussion	Urdu, Somali, Oslo	35	25–70	Pakistani, Somali
10	[17]	Islam et al	2017	Quantitative	In-person interview	New York City	12	N/A	9 females, 3 males. African/African American, South Asian
11	[18]	Pratt et al	2017	Qualitative	Focus group discussion	USA	54	18–60 +	Somali American
12	[19]	Tatari et al	2020	Qualitative	semi-structured focus group, group interviews, individual interviews	Gellerup, Aarhus, Denmark	37	27–59	Turkey, Iraq, Somalia, Lebanon, Syria, Saudi Arabia, Uzbekistan, Morocco, Pakistan, Vietnam
13	[20]	Jong et al	2021	Qualitative	Group intervention and evaluation	Scotland	18	25–65 +	Arab, Asian
14	[9]	Badre-Esfahani	2021	Qualitative	Focus group interview	Denmark	17	< 23–40 +	Turkish, Somali, Lebanon/Palestine, Iraqi, Morocco, Tunisia
15	[2]	Azhar et al	2022	Quantitative	Survey questionnaire	New York City	421	40–75	South Asian, Middle Eastern, Southeast Asian, African
16	[21]	Alam et al	2022	Quantitative	Internet-based survey	Queensland, Australia	148	20–75	Indian, Pakistani, Sri Lankan
17	[22]	Harper et al	2022	Quantitative	Survey questionnaire	Michigan, USA	893	30–65	Arab American
18	[23]	Khalid et al	2022	Qualitative	Interview	Calgary, Canada	8	26–55	South Asian
19	[24]	Alam et al	2022	Quantitative	Internet-based survey	Queensland, Australia	148	20–75	Indian, Pakistani, Sri Lankan, Other (Nepalese, Bengali, Indo-Fijian)

### Thematic analysis of CCS barriers

Based on the social-ecological model, CCS barriers were divided into four categories: individual, relationship, community, and social barriers [25]. Barriers in the individual category included sociodemographic factors, economic, language, cognitive, and emotional reactions. The relationship barrier was lack of time. Community barriers included the healthcare system, whereas culture and religion were considered social barriers. Barriers identified in each article are presented in Additional file 1. The themes of the identification results are summarized in Table 3.

**Table 3:** Thematic division

Theme	Subtheme	Barriers	N	References
Individual	Sociodemographic factors	Age groups	1	15
		Low education	4	2,3,15,19
		Single	3	2,9,17
	Economic barriers	Low income	2	1,9
		No health insurance	5	1,4,10,16,17
		Costs	1	6
		Length of stay/ immigration status	3	3,10,17
		Not employed	1	19
	Language barriers	Communication difficulties	8	1,3,9,10,12,15,16,19
	Cognitive barriers	Lack of knowledge	6	5,7,9,10,12,14
		Misunderstanding	2	7,11
		Absence of symptom	5	5,10,11,12,13
		Low perceived risk	6	4,5,7,9,10,16
		Lack of awareness	3	6,10,13
		Unfamiliar with CCS/ no information	8	2,6,9,10,12,14,16,18
	Negative feeling	Embarrassment/shame	8	1,4,5,10,13,14,16,19
		Fear of cancer	5	5,6,7,9,12
		Uncomfortable	4	4,9,13,14
		Pain	6	4,5,6,9,11,13
		Too stressful	1	13
		Afraid of the test/ bad news	2	9,12
	Affective barriers	Ignoring CCS invitation	1	14
		Influence of prior screening experiences	2	12,14
Relationship	Work and family	Lack of time because of work, childcare, and home duties	3	7,10,16
Community	Healthcare system	Sex of the physician	8	4,6,7,8,9,11,13,15
		Preference for same ethnic group physician	2	6,15
		Preference for Muslim providers	1	15
		Having no family physician/no primary care	1	8
		Distrust	5	4,7,8,9,12
		The procedure	2	5,12
		Location	3	3,5,16
		Practical barriers/long wait times	3	5,6,9
Societal	Norm	Stigma	3	7,9,10
		Muslim norms	1	4
		Taboos related to female genitalia/sexuality	1	14
		Female circumcision	3	9,12,14
	Religion	Health problems are a punishment from God	3	3,9,15
		Modesty	4	1,7,12,14
		Residing in Muslim communities of smaller size	1	3
		Being born in a Muslim-majority country	1	8
		Becoming ill and dying is the will of Allah	6	7,9,10,11,12,15

#### Theme 1: individual barriers to CCS

##### Sociodemographic factors

Low education and being single were considered substantial barriers to CCS. The results showed that immigrant Muslim women in Australia with low levels of education tended to have no knowledge of cervical cancer; therefore, they had a high probability of not attending the screening test [24]. In contrast, women in the US with a lower educational level than a bachelor's degree were found to have a high desire to undergo CCS [2]. The results of this study highlight successful counseling to Muslim immigrant communities by community-based clinics and organizations [2]. In contrast, age of the target population is rarely mentioned as a relevant factor in the reviewed articles. Only one study conducted in the US found that age was a barrier to screening. According to this study, CCS was deemed necessary and tended to be accepted by women under 50 years of age [2].

##### Economic barriers

Low economic status is a significant barrier to screening. Lack of health insurance and immigration status were identified as barriers to CCS, as well as the cost and length of stay or immigrant status. Low economic status and lack of insurance are significant barriers among immigrant Muslim women in the US. Although many participants had health insurance, they did not undergo a cancer screening test if additional payment was required [12]. Economic difficulties were also experienced by immigrant women in a study in Oslo, which found that immigrant women directed their focus towards meeting their family's basic needs rather than their own health concerns [6]. The lack of health insurance significantly reduced the desire of immigrant women in the US and Australia to attend a screening site [21, 22]. Moreover, Islam et al. (2017) reported that immigration status and fear of being deported often acted as barriers preventing many women from undergoing screening in the US [17].

##### Language barrier

Difficulty in communicating effectively caused by inadequate language skills in host countries or English is a major barrier to screening according to many studies. Language was reported as an obstacle by immigrant women in the US when communicating with health workers, although the relationship was not significant; furthermore, the study reported that women who spoke English fluently still experienced obstacles when communicating about medical issues [12]. Language barriers were also experienced by most of the participants in Queensland, Australia, and European states such as Denmark and Norway, where limited language skills made it difficult for women to access health services or health-related information, including CCS [6, 9, 19].

##### Cognitive and affective barriers

The main cognitive barrier is that many immigrants are unfamiliar with CCS. One obstacle faced by immigrant Muslims in the US limited access to information regarding CCS in their communities; consequently, they received insufficient information and did not understand the importance of the examination [17]. However, in Denmark and Norway, the lack of information was a result of the fact that information on CCS was only available in Norwegian [6, 19]. The insufficient information was primarily attributed to inadequate information received from health workers, followed by low perceived risks and lack of knowledge. Additionally, the absence of symptoms and lack of awareness are barriers to CCS [8, 14, 16, 26]. Conversely, affective obstacles are also a challenge to undergoing CCS and include ignoring invitations for CCS and the influence of previous screening experiences [9, 19].

##### Negative feeling

Most participants believed that CCS was embarrassing or shameful. Muslim women in the US reported that they felt embarrassed when discussing CCS because it involves sensitive areas of the body, which makes them reluctant to attend the examination site [12, 17]. Immigrant Muslim women from different ethnic backgrounds in European countries and Australia also described feeling shy when discussing CCS. This was also the experience of some Indian women and black women who perceived this as a barrier to screening, particularly among the older generation who feel that their body is a private and sensitive area [9, 20, 21]. Moreover, most women in the US, Canada, London, Scotland, and Dubai stated that pain during the CCS procedure and fear of cancer were major factors contributing to low CCS rates, followed by discomfort during the procedure [3, 6, 14, 18, 20, 26]. Fear of test results or bad news was also considered a barrier to CCS [6, 9, 19, 20].

#### Theme 2: family and work-related barriers to CCS

The majority of participants in the US and Australia mentioned lack of time due to work, childcare, and home duties as a challenge to undergoing screening [16, 17, 24]. Many women from low socioeconomic backgrounds earn their income from hourly wages; hence, taking time off to visit a healthcare provider for screening tests leads to a loss of income [16]. Moreover, most of the key participants also reported that sociocultural gender norms regarding women's roles were also a barrier. According to the respondents, women are expected to prioritize the health of their families above their own. This is because the existing culture has shaped the perception that women prioritize their families over themselves [17]. However, Islamic religious rules oppose spatial injustice based on sex and do not curb women's freedom to move and choose activities according to their passion. If women choose not to work and stay at home to care for their children rather than combine work and family, it is their right [27].

#### Theme 3: community barriers to CCS

##### Healthcare system

Not having a female physician was the most frequently identified barrier in the literature. Most immigrant Muslim women from the US, Canada, Scotland, Norway, and Dubai expressed their desire to be served by a female doctor [2, 3, 6, 7, 14, 16, 18, 20]. Some women in Canada and the US also stated a preference for a physician of the same ethnic group and healthcare providers with the same religion as Muslim when discussing screening tests and undergoing examinations [2, 3]. Distrust, complicated procedures, lack of accessibility to health care centers, and long waiting time are also challenges to regular screening tests [6, 7, 14, 16, 19, 26].

#### Theme 4: societal barriers to CCS

##### Norm

Studies found that there is a stigma associated with CCS among women in the US and Norway [14]. Some Muslim women in Dubai stated that Muslim norms in society regarding CCS were an obstacle to attending CCS [14]. Moreover, in European regions such as Norway and Denmark, discussions about the subject of female genitalia or sexuality are taboo and cultural practice of female circumcision, which remains a cultural practiced were considered an obstacle to undergoing CCS [6, 9, 19].

##### Religion

The main obstacle for CCS regarding religion is modesty, whereby an unmarried Muslim woman is seen as someone who is not yet sexually active, because sexual relations before marriage are prohibited in Islam. This is written in the Qur’an [9, 12, 16, 19]. Furthermore, most Muslim communities in the US, Denmark, and Norway believe that becoming ill and dying is the will of Allah, and their health problems are a punishment from God. These perceptions are barriers to Muslim women undergoing CCS [2, 6, 13, 16–19]. Research in Canada found a significant relationship between individuals born in a Muslim-majority country and a low desire to undergo CCS [7]. However, some immigrant Muslim women in the US reported that living in a country in which Muslims are a minority is an obstacle to undergoing CCS [13].

## Discussion

This scoping review examined the current literature on the barriers to CCS faced by Muslim immigrant women. The findings were obtained from several different countries. This review identified a range of barriers to CCS uptake that were specific to immigrant Muslim women and were reported in studies ranging from 2012 to 2022. The barriers affecting low CCS uptake among immigrant Muslim women were clustered into four themes: individual-level, relationship-level, healthcare system-related, and sociocultural barriers.

### Individual-level barriers

Women with low education levels and limited knowledge are less likely to undergo CCS [2, 6, 13, 16, 17, 26]. Lack of knowledge often leads to misunderstandings [16, 18] and women who are unaware of their reproductive health status ignore the risks that may impact their reproductive health [3, 6, 8, 14, 26]. The language barrier faced by Muslim immigrant women also affects their knowledge because it affects their ability to understand information regarding CCS [2, 12, 13, 19, 24]. Most women preferred information letters written in their native language. One study reported that having an interpreter staff would help women better understand the disease, which could increase their participation in CCS [6]. Another study found that, as many immigrant Muslim women speak other languages ​​at home, it would be better if the explanation of the CCS procedure were provided in a language they could easily understand as this would help educate them better regarding the importance of screening [2].

Salman (2012) identified economic status and lack of insurance as barriers to undergoing CCS [12]. Several studies reported that some Muslim women prioritize their family's economic concerns over their health problems and sought health care only when they felt ill or had health problems; furthermore, those without insurance were unlikely to attend CCS [12, 14, 22, 24]. In general, immigrants are less likely to undergo CCS; however, the limited social networks and the lack of available support, which are unique to Muslim women, represent considerable obstacles to CCS [17]

Regarding negative attitudes and readiness for CCS, embarrassment, shame, and pain were the biggest challenges for immigrant Muslim women [8, 9, 12, 14, 21, 26]. One study found strong resistance to the need to expose the female genitalia during screening and stated that discussing or participating in the screening test itself caused embarrassment [17]. Nevertheless, Muslims believe it is important to take responsibility and care for their health, and they also emphasize that this teaching can help overcome barriers to screening, such as embarrassment and shyness (22). Cervical screening was frequently described as a painful and uncomfortable procedure, particularly during the first experience [26]. Participants who were unsure about whether CCS was painful were less likely to undergo the screening than those who assumed the exam was not painful [21]. However, some women expressed a positive desire to have a checkup as soon as possible, indicating that it was easier, less painful, and shorter to undergo a gynecological examination after delivery [19].

Several studies have described the fear of cancer or worry regarding test results as factors discouraging women from attending screening [3, 6, 19, 26]. The perception that cancer cannot be cured, caused by misinformation regarding screening in the community, and that being diagnosed with cancer is similar to receiving a death sentence, scares many participants [6].

### Work and family barriers

Women spend more time on household chores, caring for spouses and children, and working longer hours, making it difficult to make time to visit screening centers. Considering the lack of time, rapid checkups at easily accessible locations may be the key to increasing screening rates [8, 16, 17, 24].

### Specific needs for healthcare providers

Owing to religious and cultural beliefs, many Muslim women feel uncomfortable when discussing CCS issues, particularly with male physicians [3, 14, 16]. Many Muslim women have a preference for female and Muslim healthcare providers, although in certain special circumstances, for example, in an emergency case, some say it is acceptable and understandable to be attended to by non-Muslims or male doctors [18]. Perceived difficulty in accessing female healthcare service providers is a significant barrier to CCS. Providing easy access to female reproductive health service providers for all immigrant Muslim women is a challenge; however, developing screening programs that incorporate cultural values, particularly in areas populated by Muslim immigrants, could offer an effective solution.

As distrust of medical care is also a potential barrier to health screenings, healthcare providers need to be trusted to provide care that respects the cultural backgrounds of Muslims in areas with large Muslim immigrant populations. Additionally, the provision of health screening by Muslim physicians may improve the CCS uptake rate among Muslim women [19].

### Barriers related to the Islamic view of women

Muslim women believe that CCS should not be performed until a woman is married, stemming from concerns regarding modesty. Many women expressing their views on screening tests highlighted the modesty barrier, encompassing considerations concerning the timing of the tests and who should undergo testing. The fact that CCS requires pelvic examination and vaginal exposure is a major challenge for Muslim women. This is unacceptable to many Muslim women before marriage because not only is the exposure considered highly embarrassing, but it is also considered to jeopardize virginity. Muslim women are concerned that the test will seriously affect virginity. Therefore, it is believed that testing can only be performed when the woman is married [19].

In addition, stigma against cervical cancer from society was cited as a CCS barrier. Muslim females associate cervical cancer with sexual activity with multiple partners, which is not accepted by Muslims, and some believe that those who do not engage in such activity should not undergo screening [17].

### Religion barriers

Religion-related factors influence CCS uptake among women born in Muslim-majority countries. Religion plays a huge role in the decision to undergo screening. Many Muslim women state that the possibility of developing cancer is in God's hands, which is the reason for refusing to undergo screening [16]. Some Muslim women feel that sickness and dying are God's will and letting the disease run its course is the right thing according to God's destiny. Conversely, others feel that health care and faith are linked, and it is part of their belief to continue seeking the best treatment and health care available to maintain the health that God has given them. This perspective involves seeing how God acts via doctors and medicine as intermediaries as a fully integrated experience of faith and medical science working together [18]. Ultimately, the success of treatment is acknowledged as being in the hands of God, but seeking treatment is undertaken in accordance with Islamic faith.

Muslims of various ethnicities present their own challenges in terms of health care, but their beliefs when facing them remains the same based on their religious values. Healthcare workers need to pay attention to and be aware of Islamic cultural values and be able to accommodate these values in practice. In hospitals, sex-appropriate care providers are prioritized. Therefore, an increased availability of female doctors within the health service will increase the interest of Muslim women in attending the examination site. Moreover, educational approaches aimed at disseminating information regarding cervical cancer and CCS can be implemented by reaching out to Muslim immigrant communities. Involving the imam of the mosque or community leader in health education may also have a positive effect on increasing screening coverage rates among Muslim immigrants.

### Limitations

Owing to restrictions on English research articles in the initial search stage, we were unable to search non-English databases and websites. However, we consider that this did not have a major impact on the review results because of the comprehensiveness of the search. Additionally, this study excluded populations such as non-Muslim immigrants, refugees, and undocumented and temporary immigrants. Therefore, our ability to extrapolate the study findings to this group is limited. Nonetheless, this analysis of the findings derived from several countries identifies the differences in CCS obstacles faced by immigrant Muslim women. Research involving many countries will provide a better picture of the obstacles faced by Muslim immigrants in obtaining cancer screening services, particularly in countries where Muslims are a minority.

## Conclusion

Cervical cancer can be prevented and treated if detected early using screening tests. Muslim immigrant women have low screening coverage rates because of many underlying barriers. Access to health care centers and CCS among Muslim immigrant women is challenging. Dissemination of information by health workers is needed to increase awareness of CCS and access to CCS service points among immigrant Muslim women. Physician recommendations to attend CCS also play an important role. The barriers summarized in this research can help healthcare providers, governments or policymakers, and researchers improve the well-being and health of this population by reducing perceived barriers to CCS, thereby increasing screening coverage rates. Future research focusing on obstacles to CCS should be conducted in different countries to provide a comprehensive overview and comparison of the perceived barriers to CCS related to cultural differences. Health service providers can increase screening coverage rates by adjusting screening programs according to the cultural and religious beliefs that prevail in a region or country.

## Data Availability

All data generated or analyzed during this study are included in this published article.

## Abbreviation

CCS: Cervical cancer screening
